# PROTOCOL: Recreational book reading for promoting cognitive functioning and emotional well‐being in older adults: A systematic review

**DOI:** 10.1002/cl2.1117

**Published:** 2020-10-06

**Authors:** Jorien Laermans, Hans Scheers, Philippe Vandekerckhove, Emmy De Buck

**Affiliations:** ^1^ Centre for Evidence‐Based Practice, Belgian Red Cross Mechelen Belgium; ^2^ Belgian Red Cross Mechelen Belgium; ^3^ Department of Public Health and Primary Care Faculty of Medicine, KU Leuven Leuven Belgium

## 1 BACKGROUND

### 1.1 Description of the condition

The world's population is growing older. According to a 2015 United Nations report (United Nations, Department of Economic and Social Affairs, & Population Division, 2015), the number of people aged 60 years or over is estimated to grow from 900 million (12.3%) in 2015 to 1.4 billion (16.5%) in 2030. This global ageing phenomenon challenges researchers and policy‐makers to investigate the factors that can promote healthy ageing, in order to alleviate the corresponding increasing socioeconomic costs. Indeed, although greater longevity comes with benefits for individuals (e.g., prolonged working life, chances for second careers), families (e.g., financial support, participation in caring for children) and society (e.g., sharing wisdom and experience, contributing to the labour force), population ageing and growth in the number of persons at very advanced ages not only creates concern about poverty rates and fiscal sustainability of pension systems, but also puts pressure on health systems (United Nations, Department of Economic and Social Affairs, & Population Division, 2015).

There is consistent evidence that physical activity is positively associated with healthy ageing (Daskalopoulou et al., 2017). Similarly, social activities (e.g., meeting friends, volunteering, participating in social clubs, making day trips) seem to have beneficial effects, for instance on longevity (Glass, Mendes de Leon, Marottoli, & Berkman, 1999) and cognitive functioning in older adults (Kelly et al., 2017). However, the effects of leisure activities that are devoid of physical and extensive social engagement, such as book reading, remain unclear.

### 1.2 Description of the intervention

The intervention of interest in this review is paper (hard copy) book reading, e‐book reading or audiobook listening as a leisure activity (“reading for pleasure” or “recreational reading”). These interventions include lone reading, as well as reading aloud in a one‐on‐one setting, and being read to in a one‐on‐one setting. These common leisure activities may promote two aspects of healthy ageing, that is, cognitive functioning and emotional well‐being, by providing cognitive stimulation and by helping to relax and take one's mind off the worries of everyday life, respectively.

### 1.3 How the intervention might work

Reading is a multifaceted cognitive process that allows us to derive meaning from print. This is accomplished by integrating word recognition, comprehension and fluency. Similarly, the cognitive process of listening enables us to derive meaning from speech or other sounds. Although the exact neuroscientific pathways remain to be elucidated, it is clear that for the human brain, reading and listening to narrated stories entails a widespread

activation of numerous cortical areas (Buchweitz, Mason, Tomitch, & Just, 2009; Dehghani et al., 2017; Willems, 2015). Books have been shown to promote “deep reading”, a slow immersive process of thoughtful and deliberate reading which is different from superficial reading (Birkerts, 1994). Deep reading not only enhances comprehension and enjoyment of a text, but also promotes inferential and deductive reasoning, analogical skills, critical analysis, reflection and insight. This may explain why book reading leads to improvements in vocabulary, reasoning, concentration, and critical thinking skills (Cunningham & Stanovich, 1998; Stanovich, West, & Harrison, 1995). In addition, reading has been shown to improve the development of empathy, social perception and emotional intelligence (Djikic, Oatley, & Moldoveanu, 2013; Kidd & Castano, 2013). There are a number of theories that could explain the beneficial effects of book reading or listening on cognition. First of all, they are hypothesized to promote healthy aging by improving cognitive reserves. The cognitive reserve theory states that “innate intelligence as well as aspects of life experience may supply a reserve in the form of a set of skills or repertoires allowing some people to cope with progressing dementia pathology better than others” (Scarmeas & Stern, 2003; Y. Stern, 2012). It is thought that complex and stimulating experiences, such as book reading, may enhance neuronal structure and brain function by creating more efficient cognitive networks, thereby providing a protective effect against neurodegeneration or cognitive decline. A second theory, the “use it or lose it” hypothesis, suggests that a lack of stimulation in everyday life can lead to faster deterioration in cognitive function (Hultsch, Hertzog, Small, & Dixon, 1999). Both theories agree on the fact that active involvement in brain‐stimulating activities may play a role in preserving cognition or slowing down cognitive decline in old age. As cognitive decline is associated with a lack of functional independence and lower quality of life, it seems plausible that building cognitive reserve through reading or listening may safeguard the emotional well‐being of older adults as well.

In addition, reading may contribute even further to the emotional well‐being of older adults through its relaxational properties. In a survey of over 4,000 people from a representative sample in the United Kingdom, regular recreational readers reported fewer feelings of stress and depression compared to nonreaders (Quick Reads & Billington, 2015). Interestingly, reading was found to induce stronger feelings of relaxation compared to watching television or engaging with technology‐intensive activities. In addition, the study revealed that regular recreational readers had higher levels of self‐esteem and a greater ability to cope with difficult situations.

The beneficial effects of book reading on cognitive engagement and stress level reduction may, in the longer term, even contribute to longevity. A U.S. population‐based cohort study demonstrated that after control for multiple sociodemographic and health‐related covariates, both severe and mild cognitive impairment were strongly predictive of subsequent mortality among older adults aged less than 80 years (Bassuk, Wypij, & Berkman, 2000). Similarly, in a U.K. survey, poorer cognitive performance was associated with an increased risk of mortality from cardiovascular disease, stroke and respiratory disease in those aged over 60 years (Shipley, Der, Taylor, & Deary, 2008). In the latter study, the possibility of reverse causality was partly excluded by reanalysing the data after omitting individuals who died within 5 years of cognitive testing. Stress represents an imminent risk factor with a documented negative impact on the immune and cardiovascular system. A large Danish population‐based cohort of 118,410 participants demonstrated that mortality rates rose with increasing levels of stress in a dose‐response relationship, independently of multimorbidity status (Prior et al., 2016).

An example of a relevant experimental study that would allow us to ascertain the adequacy of these theories might randomly assign older adults to either a reading group (e.g., where people would be provided with a book each month by their local community library) or a nonreading group (where people would be asked not to read books for the period of study duration) and follow up on their cognition and emotional well‐being at multiple timepoints. Alternatively, a relevant observational study might for example prospectively follow a cohort of older adults and assess the association between reading habits (frequency, duration etc) and cognitive decline, emotional well‐being and/or mortality, while correcting its analyses for multiple potential demographic, socioeconomic and health‐related confounders.

### 1.4 Why it is important to do this review

In 2010, a Johanna Briggs Institute systematic review was published on the effectiveness of cognitive leisure activities, including reading, watching movies, playing board games and playing musical instruments, in preventing Alzheimer's and other dementias in older adults (C. Stern & Munn, 2010). Out of the seven included observational studies, six revealed a positive association between participating in cognitive leisure activities in late life (+65 years) and a reduced risk of developing Alzheimer's and other dementia types. Moreover, the results showed that some individual leisure activities, such as reading, may be more effective than others.

A more recent literature review commissioned by The Reading Agency, aiming to collate and summarize research findings relating to nonliteracy outcomes of recreational reading, further linked recreational reading to changes in stress levels and relaxation, health literacy, and improvements in depression and dementia symptoms (The Reading Agency, 2015).

As these existing reviews have highlighted the potential beneficial effects of reading on Alzheimer's and other types of dementia only (C. Stern & Munn, 2010) or did not specifically focus on the impact on older adults (The Reading Agency, 2015), a systematic collection, extraction and analysis of quantitative data on the effectiveness of book reading on cognitive functioning and emotional well‐being of older adults is warranted.

Preparing for the economic and social shifts associated with an ageing population is an essential task for the 21st century policy‐maker. Governments should look into innovative but sustainable ways to promote healthy ageing. Reviews that study the effects of common and low‐threshold leisure activities, such as book reading, on the cognition and emotional well‐being of older adults, may provide useful information in creating these frameworks. In this way, policy‐makers can make evidence‐based decisions on where to focus their efforts in dealing with global ageing.

## 2 OBJECTIVES

By systematically searching for individual studies, this review will answer the following research question:

What is the effect of book reading on cognitive functioning and emotional well‐being in older adults?

## 3 METHODS

### 3.1 Criteria for considering studies for this review

#### 3.1.1 Types of studies

Since we will apply quite specific criteria at the level of population and intervention, we will include a broad range of study designs to ensure that the systematic review is as inclusive as possible.

Studies using an experimental design (randomized controlled trials, quasi‐ or nonrandomized controlled trials, controlled before and after study or controlled interrupted time series) will be included. In addition, we will also include studies using an observational design (cohort study, case‐control study, controlled before and after study, controlled interrupted time series, cross‐sectional study), as we anticipate that they will provide the majority of the available evidence. Observational studies will be included regardless of the fact if they adequality controlled their analyses for potential confounding factors (e.g., early‐life participation in reading activities, degree of book availability due to socio‐economic status). However, the impact of this decision will be further investigated during Data collection and analysis (see below).

Other study designs such as case series, narrative reviews and nonoriginal studies such as editorials, book reviews, commentaries, and letters to the editor, will be excluded.

In addition, qualitative studies will not be included in this review.

#### 3.1.2 Types of participants

Studies in community‐dwelling and institutionalized older adults (≥60 years of age) will be included. In this systematic review, the term “community‐dwelling” refers to individuals who live in a private residence (e.g., their own house or apartment, the house or apartment of one of their family members). “Institutionalized” refers to individuals residing in a nursing home or another assisted living facility.

Studies that also include younger adults (<60 years of age) will only be included if: (a) they report the results separately for ≥ 60‐year‐olds, or (b) they specifically define the population as “older adults” or “elderly” and the average age of the study participants is or exceeds the age of 60.

This review will include studies in older adults with normal or mildly impaired cognition at baseline, as determined using the Mini‐Mental State Examination (MMSE; Folstein, Folstein, & McHugh, 1975), the standard version of the Mini‐Mental State Examination Second Edition (MMSE‐2:SV; Folstein, Folstein, White, & Messer, 2010) or the Montreal Cognitive Assessment (MoCA; Nasreddine et al., 2005):

- Normal cognition: scoring ≥25 in the MMSE/MMSE‐2:SV or ≥25 in the MoCA;
- Mildly impaired cognition: scoring 21‐24 in the MMSE/MMSE‐2:SV or 18‐25 in the MoCA, including patients suffering from:
  ￮ Mild cognitive impairment (MCI), who still function independently or nearly so in their daily lives in a manner that is indistinguishable from the past (Petersen et al., 1997).
  ￮ Mild dementia, who experience significant difficulties in daily life that interfere with independence. In contrast to more severe forms of dementia where basic activities of daily living are compromised, patients with mild dementia retain independence in simpler activities (Knopman & Petersen, 2014).

Studies that do not mention cognition levels of the participants will be included, as we assume they will have recruited “normal, healthy” older adults. Studies that include mixed populations consisting of older adults with normal cognition ánd older adults with mildly impaired cognition will be included. Studies that also include older adults with moderate or severe cognitive impairment (scoring < 21 in the MMSE or <18 in the MoCA) will be excluded from the review, unless they report the results separately for the participants with normal cognition and/or mildly impaired cognition.

#### 3.1.3 Types of interventions

Interventions for this systematic review will include any frequency and any duration of lone recreational paper (hard copy) book reading, e‐book reading or audiobook listening. In addition, interventions where an older adult reads aloud to another adult or child, or where an older adult isbeing read toby another adult or child, will also be included. Interventions that are accompanied by extensive social interaction or engagement, such as book reading clubs and group reading, will be excluded.

As books are considered to engage readers’ minds more than newspapers, periodicals and magazines, the latter three reading materials will be excluded from the review. In addition, studies looking at the combined effects of reading books and reading periodicals/magazines/newspapers will be excluded. Studies that do not specify the type of reading materials will also be excluded. In addition, interventions where book reading is offered as a therapeutic treatment (i.e., bibliotherapy) will be excluded.

Within experimental studies, the effect of book reading will be compared to no paper or e‐book reading, or no audiobook listening. For observational studies, the outcomes (see below) of older adults who read paper books, read e‐books or listen to audiobooks, will be compared to those of older adults who did not participate in these activities.

#### 3.1.4 Types of outcome measures

Studies will be included if they have measured the effect of book reading on one or more of the following primary or secondary outcomes.

Studies will not be excluded solely on the basis of reporting of outcome data. To this end, we will contact the authors to ascertain whether the data for our outcomes of interest are unavailable due to lack of measurement or lack of reporting.

##### 3.1.4.1 Primary outcomes

The primary outcomes of interest are cognitive functioning and emotional well‐being.

Studies that have measured (a decline in) cognitive functioning will be included, regardless of the measurement instrument used. Measuring instruments include, but are not limited to:

- The Mini‐Mental State Examination (MMSE);
- The standard version of the Mini‐Mental State Examination Second Edition (MMSE‐2:SV);
- Montreal Cognitive Assessment (MoCA).

Studies that have measured clinician‐rated and self‐reported mood and notions of emotional well‐being (e.g., life satisfaction, quality of life, anxiety, subjective happiness, depressive symptom experiencing) will also be included. Measuring instruments include, but are not limited to:

- The Psychological Wellbeing Scale (Ryff, 1989) or its shortened version (Ryff & Keyes, 1995);
- The 22‐item General Well‐being Schedule (Dupuy, 1977);
- The Life Satisfaction Index A (LSIA; Neugarten, Havighurts, & Tobin, 1961);
- The CASP‐19 Quality of Life Scale (Hyde, Wiggins, Higgs, & Blane, 2003);
- The EuroQol 5 Dimensions scales ((EQ‐5D); EuroQol Group, 1990);
- The Mood and Anxiety Symptom Questionnaire (MASQ; Clark and Watson, 1991);
- The 21‐item Beck Depression Inventory (BDI; Beck, Ward, Mendelson, Mock, & Erbaugh, 1961) or its shortened 13‐item version (Beck and Beck, 1972).

This systematic review will be comprehensive regarding the timing of these measurements. In other words, we will include:

- Studies that have assessed an outcome once during the post‐intervention period (immediately after the intervention or in the longer term).
- Studies that have assessed the same outcome multiple times during the post‐intervention period (e.g., immediately after the intervention and 6 months later).
- Studies that have assessed the same outcome before the start of the intervention and post‐intervention.

##### 3.1.4.2 Secondary outcomes

The secondary outcome of interest is mortality/survival.

### 3.2 Search methods for identification of studies

A comprehensive search for eligible published and unpublished studies and reports will be performed to reduce the risk of publication bias and identify the best available evidence. No date, location or language restrictions will be placed on the searches or included studies.

#### 3.2.1 Electronic searches

##### 3.2.1.1 Electronic databases

The following databases will be searched from inception to present:

- The Cochrane Library (Cochrane Database of Systematic Reviews and Cochrane Central Register of Controlled Trials);
- MEDLINE (PubMed interface);
- Embase (Embase.com interface);
- The Education Resources Information Center (ERIC; OVID interface);
- Web of Science Core Collection.

On the basis of previously published relevant papers and our selection criteria, a sensitive search strategy will be developed by JL and EDB, researcher and senior researcher at the Centre for Evidence‐Based Practice, where evidence‐based guidelines and systematic reviews are developed on a daily basis.

The strategy will be tailored to each specific database and will comprise both index terms (when relevant; e.g., MeSH terms, Emtree terms) and free text words (in title or abstract), with attention to possible synonyms, spelling variants, and correct use of truncation and proximity operators. Search filters will not be used, as they may prevent the retrieval of relevant papers.

De‐duplication of the references will be done using the EndNote reference management software (EndNote, 2013). All searches and search dates will be documented.

Below, the search strategy for MEDLINE (PubMed interface) is provided:

1. “Aged”[Mesh] OR aged[TIAB] OR elderly[TIAB] OR “Retirement”[Mesh] OR retirement[TIAB] OR retired[TIAB] OR pension[TIAB] OR pensioner[TIAB] OR pensioning [TIAB] OR pensioned[TIAB] OR “old people”[TIAB] OR “older adult”[TIAB] OR “older adults”[TIAB] OR “older people”[TIAB] OR “old age”[TIAB] OR “older age”[TIAB] OR “Geriatrics”[Mesh] OR geriatric[TIAB] OR geriatrics[TIAB] OR ((resident[TIAB] OR residents[TIAB]) AND (“retirement home”[TIAB] OR “retirement homes”[TIAB] OR “nursing home”[TIAB] OR “nursing homes”[TIAB]))
2. “Books”[Mesh] OR book[TIAB] OR books[TIAB] OR audiobook[TIAB] OR audiobooks[TIAB] OR e‐book[TIAB] OR e‐books[TIAB] OR ebook[TIAB] OR ebooks[TIAB] OR “Leisure activities”[Mesh] OR leisure[TIAB] OR pleasure[TIAB] OR recreational[TIAB] OR “Literature”[Mesh] OR “Libraries”[Mesh:NoExp] OR library[TIAB] OR libraries[TIAB] OR poetry[TIAB] OR novel[TIAB] OR novels[TIAB]
3. “Reading”[Mesh] OR read[TIAB] OR reads[TIAB] OR reading[TIAB] OR (watching[TIAB] AND (TV[TIAB] OR television[TIAB] OR movie[TIAB] OR movies[TIAB])) OR (listening[TIAB] AND (music[TIAB] OR radio[TIAB])) OR dancing[TIAB] OR gardening[TIAB] OR ((game[TIAB] OR games[TIAB]) AND (play[TIAB] OR plays[TIAB] OR playing[TIAB])) OR gaming[TIAB] OR puzzle[TIAB] OR puzzles[TIAB] OR puzzling[TIAB]
4. 1‐3 AND

##### 3.2.1.2 Grey literature sources and handsearching

We will consult the following sources of grey literature, and search the websites of organizations devoted to the specific topics of reading/leisure and ageing, to identify relevant unpublished studies and reports:

- Grey literature:
  - Grey literature repositories:
    ▪ Grey Literature Report (www.greylit.org);
    ▪ OpenGrey (www.opengrey.eu);
    ▪ ClinicalTrials.gov (clinicaltrials.gov);
    ▪ International Clinical Trials Registry Platform of the World Health; Organization (ICTRP, apps.who.int/trialsearch/Default.aspx);
  - Other sources of grey literature:
    ▪ Google Scholar (scholar.google.com).
- Reading and leisure activities:
  - EU Read (www.euread.com/research);
  - The Reading Agency UK (readingagency.org.uk/adults/impact/);
  - The Reader (www.thereader.org.uk/research);
  - Dutch Reading Foundation (Stichting Lezen Nederland; www.lezen.nl);
  - Hill Strategies Research Inc., “Statistical Insights on the Arts” series (www.hillstrategies.com/);
  - World Health Organization (WHO; www.who.int/en/).
- Ageing:
  - Age UK (www.ageuk.org.uk/our-impact/policy-research/publications/);
  - Centre for Ageing Better (www.ageing-better.org.uk/publications);
  - International Longevity Centre UK (ILCUK, ilcuk.org.uk/reports/);
  - WHO Ageing and life‐course Program (www.who.int/ageing/data-research/en/);
  - National Ageing Research Institute (NARI) in Victoria, Australia (www.nari.net.au/publications/overview-about-publications).

In compiling this list of organizations, the following criteria were applied:

- On their websites, the included organizations should explicitly state or show that they perform or bundle evaluations or reports on the effectiveness of (reading/ageing) interventions. In addition, these evaluations or reports should be readily available on their websites.
- University research groups were not included, as we expect them to publish their work in peer‐reviewed journals.

#### 3.2.2 Searching other resources

##### 3.2.2.1 Other reviews

The reference list of the systematic reviews on the effectiveness of cognitive leisure activities (C. Stern & Munn, 2010) and on the nonliteracy outcomes of recreational reading (The Reading Agency, 2015), as well as those of the systematic reviews identified using the search mentioned above, will be scanned for relevant references.

##### 3.2.2.2 Reference lists

The reference lists of included references will be searched.

##### 3.2.2.3 Contacting experts

This review will be conducted in close collaboration with the Social Care Department of Belgian Red Cross. This Department runs a Care Library, a project in which need‐adapted library materials are provided to hospitals, residential care centres, rehabilitation centres… Furthermore, the review team will also receive content support from an external panel of social care experts (Vonk3 research centre of Thomas More University, Expertise centre Dementia Flanders, residential care centres, Public Centre for Social Welfare, Christian health insurance fund). These experts will be contacted to help identify other relevant studies.

### 3.3 Data collection and analysis

#### 3.3.1 Selection of studies

Study selection will be performed independently and in parallel by two evidence reviewers (JL and HS) in EndNote. In a first phase, titles and abstracts of the references identified by the search will be screened. Full texts of potentially relevant papers will be retrieved, and references that meet the selection criteria will be included for further analysis. Any relevant retraction statements and errata will be examined. In addition, relevant conference abstracts identified through the above‐mentioned searches will be included. Studies that meet the selection criteria and had the outcomes of interest measured, but do not report these outcome data, will be included and described in the Results section of the full systematic review.

Any discrepancies between the two reviewers will be resolved by consensus, and in case of disagreement a third reviewer will be involved (EDB). A PRISMA study selection flow chart will be provided (Moher, Liberati, Tetzlaff, Altman, & The PRISMA Group, 2009) and a table of “Characteristics of excluded studies” will be presented in the full systematic review.

#### 3.3.2 Data extraction and management

Data concerning the year in which the study was reported, the setting, the study design, and the basic characteristics of the study participants, interventions, and outcome measures will be independently extracted by the two reviewers. To ensure consistency in the data collection process, a standardized and piloted data collection form will be used (see Appendix A).

By documenting all eligible available outcome measures in the Characteristics of included studies table, the two reviewers will be able to assess the potential for multiplicity of outcomes within the same study and handle them appropriately, following the guidance of the Cochrane Handbook for Systematic Reviews of Interventions (McKenzie et al., 2019).

If multiple methods are used to measure the same outcome within the same study, the reviewers will select the most relevant measures for analysis using the following decision rules:

- Outcome measures obtained via validated formal scales are more relevant than those obtained via a single‐item question or non‐validated scales;
- Preference will be given to the measure used most frequently in the included studies;
- Self‐reported and clinician‐reported ratings are considered equally important. Therefore, if a study reports both, one self‐reported and one clinician‐reported rating will be extracted.

If a single study has measured the same outcome at multiple time points, the reviewers will extract data from one short‐term time point (≤1 month after the intervention has ended), one intermediate‐term time point (>1 and ≤6 months after the intervention has ended) and one long‐term time point (>6 months after the intervention has ended).

If a single study only reports a composite measure of two or more of the outcomes of interest, the composite will be extracted and analysed.

If a study both contains data on overall scale findings, but also on the different dimensions addressed by the scale, only the overall scale results will be extracted.

During extraction, special attention will be paid to ensure that multiple reports of the same study are not treated as multiple studies. Should a study contain multiple intervention arms, the reviewers will only extract data on the intervention and control groups that are eligible to this review. Should a multiarm study report multiple relevant intervention arms, the findings from the different arms will be reported and analysed separately.

Experimental and observational studies will be extracted and analysed separately.

For dichotomous outcomes, the number of events and the number of participants in each (intervention or control) group will be extracted. Odds ratios or risk ratios (both crude and adjusted ratios, if available) will be extracted, along with their 95% confidence intervals (CIs) and *p* values.

For continuous outcomes that can be assumed normally distributed, we will extract means, standard deviations (or information to estimate standard deviations), and the number of participants in each group. For skewed continuous data, medians, ranges, and *p* values of nonparametric tests will be extracted.

In case of controlled before and after studies, mean or median change‐from‐baseline scores will be extracted, or computed by the reviewers if all necessary data are available. If change scores are not available or cannot be computed, post‐intervention values will be extracted by the reviewers.

Any discrepancies between the two reviewers will be resolved through discussion or consulting other review co‐authors.

#### 3.3.3 Assessment of risk of bias in included studies

Individual studies will be assessed for risk of bias, independently by the two reviewers. For randomized controlled trials, the Cochrane Risk of Bias tool will be used to identify the methodological quality and potential shortcomings therein (Higgins & Green, 2011). Study quality of nonrandomized experimental and observational studies will be assessed using the Risk of Bias In Non‐randomized Studies‐of Interventions (ROBINS‐I) tool (Sterne et al., 2016).

Next, the GRADE approach will be used to assess the overall certainty of the evidence included in this review, based on the limitations in study design, imprecision, inconsistency, indirectness, and publication bias (Atkins et al., 2004; Schünemann, Brozek, Guyatt, & Oxman, 2013). The certainty of the “body of evidence” will be assigned, ranging from high, moderate, low to very low.

#### 3.3.4 Measures of treatment effect

Continuous outcomes will be reported as mean differences (MD) or standardized mean differences (SMD; when studies assess the same outcome, but measure it in a variety of ways, e.g., using different scales) with 95% CIs.Dichotomous outcomes will be reported as odds ratios (OR) or risk ratios (RR) with 95% CIs.

A “Summary of findings” table will be provided in the review, containing a summary of the results of all the included studies.

#### 3.3.5 Unit of analysis issues

Should we encounter a multi‐arm study, we will pay caution to ensure that the same group of participants is not included twice in a single meta‐analysis. In addition, paired data will be analysed appropriately.

#### 3.3.6 Dealing with missing data

In case of missing data, we will contact the authors at least twice to obtain these data, if correspondence details are available.

Where possible, we will calculate missing values (e.g., risk ratios, 95% CI and *p* values) from the available data, using the Review Manager 5 software (Higgins, Li, & Deeks, 2019; Review Manager, 2014). If insufficient data are available to calculate missing values, we will only analyse the available data and describe the results from the studies with missing data narratively.

In the final review, the issue of the missing data and their potential impact on the findings will be discussed in the Discussion section.

#### 3.3.7 Assessment of heterogeneity

Forest plots will be inspected to visually investigate overlap in the confidence intervals for the results of the individual studies. The chi‐squared test will be performed and the I² statistic will be calculated to quantify inconsistency across studies. For the chi‐squared test, a p‐value of 0.10 will be used as a threshold for statistical significance. An I² threshold of 60% will be adopted. However, following the guidance of the Cochrane Handbook for Systematic Reviews of Intervention (Deeks, Higgins, & Altman, 2019), care will be taken in interpreting the results, should studies be few in number or have small sample sizes.

#### 3.3.8 Assessment of reporting biases

If 10 or more studies are identified, publication bias will be assessed through visual inspection of funnel plots. If the funnel plot shows asymmetry, a formal statistical Egger test will be performed. If there is evidence of funnel plot asymmetry from a test, we will attempt to distinguish the different possible reasons for this (nonreporting biases, poor methodological quality leading to spuriously inflated effects in smaller studies, true heterogeneity, artefactual, chance) (Page, Higgins, & Sterne, 2019).

#### 3.3.9 Data synthesis

Experimental and observational studies will be analysed separately. Should cluster randomized controlled trials be included, they will be scrutinized and, if necessary, their analyses will be adjusted for clustering. If 2 or more studies are identified that have investigated the effect of the same intervention on the same outcome, and data are sufficiently available, these data will be pooled and random effects meta‐analyses will be performed due to the expected between‐study variation, using the Review Manager 5 software. The Mantel–Haenszel method and the Inverse‐Variance method will be used for dichotomous and continuous outcomes, respectively. Meta‐analysis results will be visually presented in forest plots.

Change scores and post‐intervention values will be combined in the same meta‐analysis using the MD approach, in accordance with the guidance of the Cochrane Handbook for Systematic Reviews of Interventions (Deeks et al., 2019).

Should we encounter a combination of dichotomous and continuous data for the same outcome or predictor, we will first try to resolve this issue by collecting missing data from the study authors. If it remains impossible to summarize the results from all the relevant studies in a similar way, we will report and analyse the dichotomous and continuous data separately (Deeks et al., 2019).

In case a quantitative synthesis is not possible, study findings will be synthesized alternatively. To this end, we will use one of the acceptable alternative synthesis methods and visual display methods as described in the Cochrane Handbook for Systematic Reviews of Interventions (McKenzie & Brennan, 2019).

#### 3.3.10 Subgroup analysis and investigation of heterogeneity

If substantial statistical heterogeneity is detected, heterogeneity may be explored by conducting subgroup analyses or (if at least 10 studies are included in the meta‐analysis) by conducting meta‐regression to guard against potential issues of confounding (Deeks et al., 2019). Heterogeneity may occur due to:

1. Type of reading material: there may be a difference between the effects of hard copy book reading, e‐book reading and audiobook listening. Despite the high similarity in cortical areas recruited for listening and reading comprehension processes (Jobard, Vigneau, Mazoyer, & Tzourio‐Mazoyer, 2007), several brain imaging studies have indicated that both modalities activate specific and distinct brain areas (Buchweitz et al., 2009; Cohen et al., 2002).
2. Reading frequency and duration: we hypothesize that book reading will have a “dose‐dependent” effect on cognition and/or emotional well‐being, that is, that high‐frequency reading (as categorized by the study authors for example as “frequent book reading”, or at least once or twice per week) and increased reading duration (at least 3.5 hr per week) will have more beneficial effects compared to lower‐frequency reading and shorter reading duration.
3. Socioeconomic diversity: book reading may differentially affect people depending on the current and past availability of books, and the reading habits developed during early childhood.
4. Lone reading versus reading aloud/being read to in a one‐on‐one setting: lone reading may have differential effects compared to reading that occurs in the company of another person.
5. Cognition levels at baseline: book reading may have a more pronounced effect on inhibiting cognitive decline in older adults with normal cognition levels at baseline, compared to older adults with mildly impaired cognition.

As direct analysis of more than two subgroups is not possible in the Review Manager 5 software, subgroups will be compared two by two, whether the outcome is continuous or dichotomous. *p* values will appropriately be adjusted for multiple testing.

Should post hoc subgroup analyses be conducted, we will clearly state in the review that these analyses are post hoc and exploratory in nature.

If a sufficient number of studies are identified, meta‐regression will be conducted using the R statistical software package, as this is not possible in the Review Manager 5 software.

#### 3.3.11 Sensitivity analysis

Sensitivity analyses may be performed with respect to the quality of studies to test the robustness of the meta‐analysis by assessing whether results are not influenced by the inclusion or exclusion of low‐quality studies.

## AUTHOR CONTRIBUTIONS

J. L. drafted the protocol. All authors reviewed the draft and approved the final version.

## CONFLICT OF INTERESTS

J. L., H. S., P. V. and E. D. B. are employees of the Belgian Red Cross and have no further interests to declare. One of the activities of the Belgian Red Cross is to run a Care Library, a project in which need‐adapted library materials are provided to hospitals, residential care centres, rehabilitation centres, etc.

## SOURCES OF SUPPORT

### Internal sources

- Belgian Red Cross, Belgium

This systematic review is funded by the Foundation for Scientific Research of the Belgian Red Cross.

### External sources

- No sources of support provided

## APPENDIX A DATA COLLECTION FORM

**Table:**

**Characteristics of included studies**				
Author, year, country	Study design	Population	Comparison	Remarks
**Synthesis of findings**				
Outcome	Comparison	Effect size	# studies, # participants	Reference
